# Layered Structural PBAT Composite Foams for Efficient Electromagnetic Interference Shielding

**DOI:** 10.1007/s40820-023-01246-8

**Published:** 2023-11-23

**Authors:** Jianming Yang, Hu Wang, Yali Zhang, Hexin Zhang, Junwei Gu

**Affiliations:** 1 School of Chemistry and Chemical Engineering, Anhui University of Technology, Ma’anshan, Anhui, 243032 People’s Republic of China; 2 Shaanxi Key Laboratory of Macromolecular Science and Technology, School of Chemistry and Chemical Engineering, Northwestern Polytechnical University, Xi’an, 710072 Shaanxi People’s Republic of China; 3 School of Materials Science and Engineering, Jiangsu University of Science and Technology, Zhenjiang, 212003 Jiangsu People’s Republic of China

**Keywords:** Electromagnetic interference shielding, Layered structure, Supercritical carbon dioxide foaming, Poly (butyleneadipate-*co*-terephthalate), Microcellular

## Abstract

The utilization of eco-friendly, lightweight, high-efficiency and high-absorbing electromagnetic interference (EMI) shielding composites is imperative in light of the worldwide promotion of sustainable manufacturing. In this work, magnetic poly (butyleneadipate-*co*-terephthalate) (PBAT) microspheres were firstly synthesized via phase separation method, then PBAT composite foams with layered structure was constructed through the supercritical carbon dioxide foaming and scraping techniques. The merits of integrating ferroferric oxide-loaded multi-walled carbon nanotubes (Fe_3_O_4_@MWCNTs) nanoparticles, a microcellular framework, and a highly conductive silver layer have been judiciously orchestrated within this distinctive layered configuration. Microwaves are consumed throughout the process of “absorption-reflection-reabsorption” as much as possible, which greatly declines the secondary radiation pollution. The biodegradable PBAT composite foams achieved an EMI shielding effectiveness of up to 68 dB and an absorptivity of 77%, and authenticated favorable stabilization after the tape adhesion experiment.

**Supplementary Information:**

The online version contains supplementary material available at 10.1007/s40820-023-01246-8.

## Highlights

- A layered segregated shielding network was organized in porous PBAT/Fe_3_O_4_@MWCNTs/Ag composite by scCO_2_ foaming and scraping techniques.
- The composite foam achieved an electromagnetic interference (EMI) shielding effectiveness (SE) of up to 68.0 dB and a reflectivity of as low as 23% due to the “absorption-reflection-re-absorption” shielding mechanism.
- The solid and foamed PBAT/Fe_3_O_4_@MWCNTs/Ag composites displayed superior retention (> 92%) of EMI SE even after peeling experiment of 500 times under 100 g weight pressure.

**Supplementary Information:**

The online version contains supplementary material available at 10.1007/s40820-023-01246-8.

## Graphical abstract

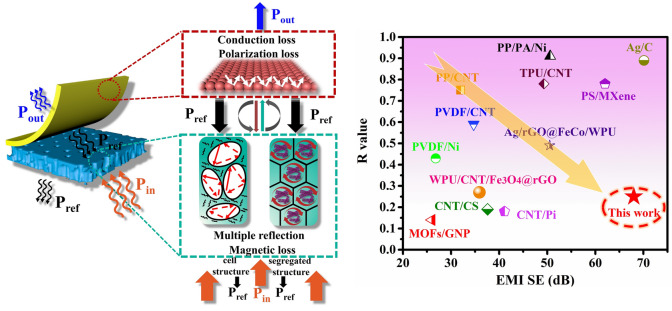

## Introduction

In contemporary society, the proliferation of portable electronic devices and the advent of 5th Generation Mobile Communication Technology (5G) have rendered electromagnetic (EM) radiation a progressively escalating environmental concern of paramount significance [1–5]. Thus, the design and exploitation of high-performance electromagnetic interference (EMI) shielding materials has become an urgent need at present [6–8]. Conductive polymer composites are widely used in EM protection due to their light weight, easy molding and anti-corrosion [9–13]. Regrettably, the rapid turnover of digital equipment in today’s landscape results in the widespread abandonment of replaced electronic devices, transforming them into “electronic waste”. Furthermore, there exists a global economic consensus emphasizing environmental friendliness and acknowledging the imperative of sustainable development. Hence, the research and deconstruction of recyclable and biodegradable EMI shielding composites is expected to be a major focus in the field of EM protection for the future.

Traditional EMI shielding composites suffer from suboptimal filler overlap, necessitating a significant filler content to attain high EMI shielding effectiveness (SE) exceeding 50 dB. Unfortunately, this often leads to adverse consequences on the mechanical properties of the composite [14–16]. Recently, a variety of novel conductive network designs such as isolated structure, double percolation structure and sandwich structure were leveraged for the sake of reducing filler content and strengthening the EMI SE of composites [17, 18]. Nevertheless, such composites mainly block EM waves by reflecting them into outer space, which is susceptible to inevitable secondary pollution [19–24]. A distinctive microwave attenuation mechanism can be achieved through the purposeful design of a layered structure incorporating magnetic nanoparticles infused with conductive fillers. When coupled with the systematic adjustment of various absorption and reflection layers, there is a promising avenue for surmounting the challenge of reconciling low reflectivity with high EMI SE.

Light weight has always been the development direction of EMI shielding composites along with the gradual integration and miniaturization of electronic components. The latest studies imply that the introduction of microcellular can not only effectively reduce the density of composite but also alleviates the impedance mismatch and enhance the attenuation of EM waves through multiple reflections and scattering inside the porous structure [25–28]. Among the many foaming techniques, supercritical carbon dioxide (scCO_2_) foaming has been extensively employed for the preparation of microcellular composites due to its environmental friendliness, operation simplicity and short molding period [29–34]. Thus, it is believed that the combination of layered and microcellular structure can be anticipated to perform a high shielding efficiency with the more favorable EM wave absorption and prevention of secondary EM radiation pollution.

Herein, we firstly fabricated poly (butyleneadipate-*co*-terephthalate) (PBAT)-Fe_3_O_4_@MWCNTs microspheres by phase separation techniques. The isolated, porous network was successfully constructed via compression molding and scCO_2_ foaming following the wrapping of nickel particles on the microsphere surface, subsequently a layered structure was established in accordance with the silver particle coating. EM waves were consumed through the magnetic loss of Fe_3_O_4_@MWCNTs nanoparticles and multi-reflection absorption of microcellular, and then reflected back into the composite foam once the microwaves arrived at the Ag layer and undergo secondary absorption procedure. The resulting composite foam demonstrates an impressive EMI SE of 68.0 dB while maintaining a low reflectivity of just 23%. This achievement can be attributed to the underlying “absorption-reflection-reabsorption” shielding mechanism, thus proposing a viable approach for the development of biodegradable EMI shielding materials characterized by minimal reflection properties.

## Experimental Section

### Materials

PBAT granules were purchased from BASF Ltd., Germany. Fe_3_O_4_@MWCNTs nanoparticles were developed by co-precipitation as described in our previous work [9]. PVA was supplied by Shanghai Junchen Chemical Technology Co., Ltd. Conductive silver paint (SPI, 05001-AB) with a silver content of 43 wt% was obtained from Huida Electronic Technology Co., Ltd., China. HCl, CH_2_Cl_2_, C_6_H_5_OH were supplied by Shanghai Aladdin Biochemical Technology Co., Ltd., China.

### Fabrication of PBAT-Fe_3_O_4_@MWCNTs and PBAT-Fe_3_O_4_@MWCNTs/Ni Microspheres

**Fig. 1:**
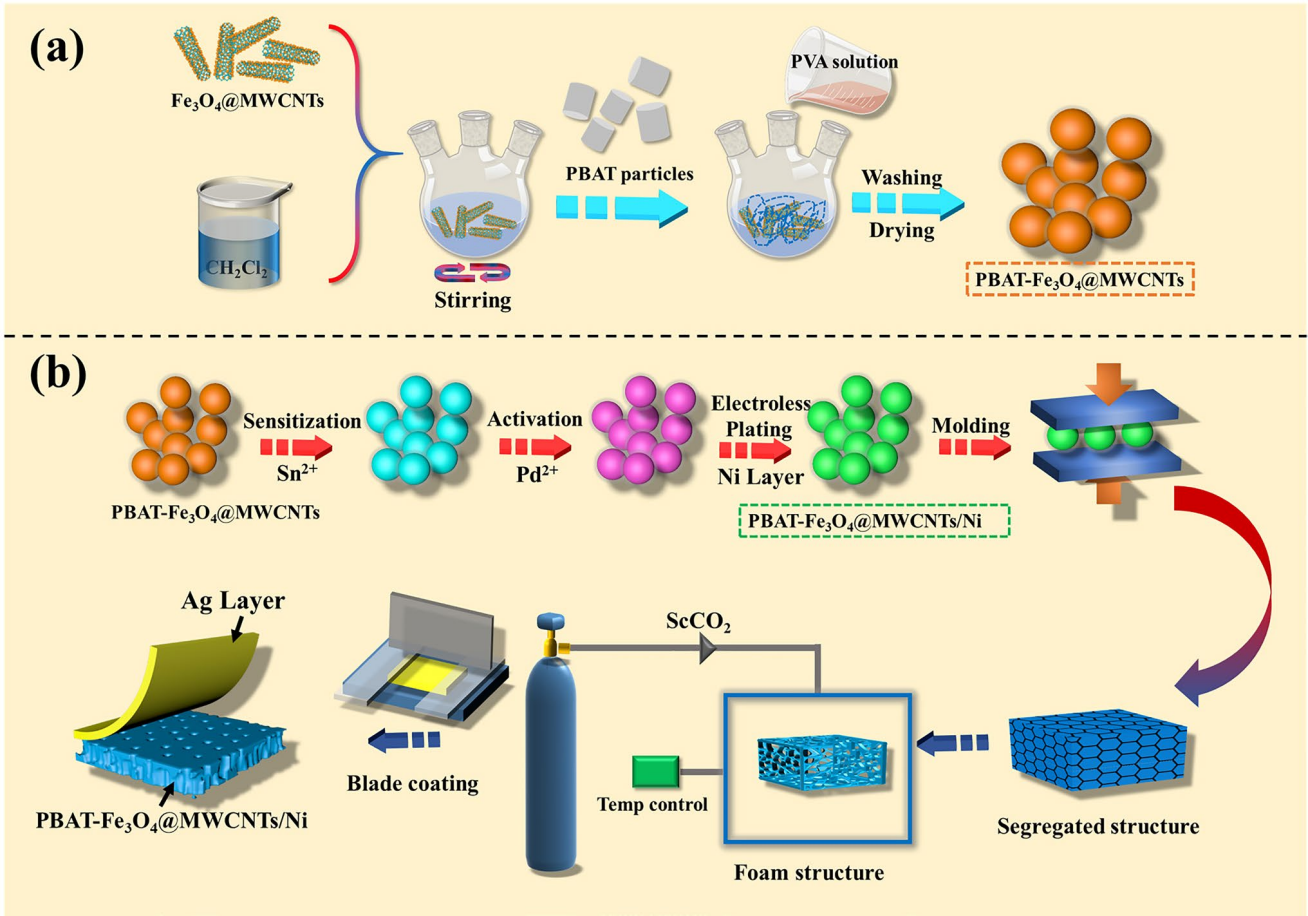
Schematics for the synthesis of **a** PBAT-Fe_3_O_4_@MWCNTs microspheres and **b** preparation of PBAT-Fe_3_O_4_@MWCNTs/Ni/Ag composite foams

PBAT-Fe_3_O_4_@MWCNTs microsphere were prepared through phase separation technique (Fig. 1a) [10, 35]. Fe_3_O_4_@MWCNTs nanoparticles were initially incorporated in CH_2_Cl_2_, ultrasonically dispersed and stirred for 1 h. Subsequently, PBAT particles were added, dissolved and continued stirring for 8 h. The final product was extracted and washed with deionized water, then dried under vacuum for 2 h and collected. PBAT-Fe_3_O_4_@MWCNTs/Ni microspheres were prepared by chemical deposition as shown in Fig. 1b. 3 g of PBAT-Fe_3_O_4_@MWCNTs microspheres were first added to 100 mL of H_2_SO_4_ solution (36 wt%), stirred for 45 min at room temperature, filtered and washed, then dispersed in the sensitized solution (consist of 100 mL distilled water, 1 g SnCl_2_·2H_2_O and 2 mL HCl) and stirred mechanically for 30 min. The sensitized microspheres were then added to 100 mL of PdCl_2_ solution (0.1 g L^−1^), mechanically stirred for 30 min, and then filtered and washed. The activated microspheres were deposited into the chemical plating solution (including 100 ml distilled water, 2 g NiCl_2_·6H_2_O and 3 g Na_3_C_6_H_5_O_7_⋅2H_2_O), and the reducing solution (containing 4 g NaH_2_PO_2_⋅H_2_O and 10 mL distilled water) was added dropping by dropping after the solution was warmed up to 85 °C with continuous stirring and reaction for 20 min. In the end, the products were repeatedly washed with deionized water and dried to obtain PBAT-Fe_3_O_4_@MWCNTs/Ni microspheres.

### Preparation of PBAT-Fe_3_O_4_@MWCNTs/Ni/Ag Composite Foams

As shown in Fig. 1b, the PBAT-Fe_3_O_4_@MWCNTs/Ni microspheres were firstly placed in a tailored mold and then hot pressed at 130 °C and 10 MPa for 10 min to acquire the isolated PBAT-Fe_3_O_4_@MWCNTs/Ni composites. The samples were transferred into a high-pressure reactor with CO_2_, and saturated for 2 h once the temperature reached 125 °C and the pressure up to 12 MPa. After that, the gas was quickly released and discharged to atmospheric pressure to achieve the foamed PBAT-Fe_3_O_4_@MWCNTs/Ni composites. For the purpose of regulating the thickness of foamed composite, the solid samples were confined in the high-pressure reactor by tailored molds and the height of foamed composite could be controlled. Finally, the conductive silver adhesive was applied to the surface of samples by the scraping method, and the curing of the silver layer was completed by keeping the solvent volatilized in a drying oven at 60 °C for 4 h, thus constituting the PBAT-Fe_3_O_4_@MWCNTs/Ni/Ag composite foams with a layered structure. Here, solid PBAT-Fe_3_O_4_@MWCNTs/Ni/Ag composites were obtained by scraping and drying on the basis of isolated PBAT-Fe_3_O_4_@MWCNTs/Ni composites.

In order to investigate the effects of Fe_3_O_4_@MWCNTs content, sample thickness and foaming treatment on the EMI shielding properties of the composites, the content of Fe_3_O_4_@MWCNTs was controlled to be 5, 10 and 15 wt% in this work, and the thicknesses of the samples were 1.8, 2.8 and 5.5 mm, respectively. For the sake of simplicity, the composites were named as “S-PFx/Ni/Ag” and “F-PFx/Ni/Ag”, where “S” stands for solid sample, “F” represents the foamed sample and “x” refers to the content of Fe_3_O_4_@MWCNTs. For example, “S-PF5/Ni/Ag” indicates a solid PBAT-Fe_3_O_4_@MWCNTs/Ni/Ag composite with 5 wt% Fe_3_O_4_@MWCNTs, and “F-PF15/Ni/Ag” denotes a foamed PBAT-Fe_3_O_4_@MWCNTs/Ni/Ag composite with 15 wt% Fe_3_O_4_@MWCNTs. The “Characterization” section was supplied in the supporting information.

## Results and Discussion

### Characterizations and Morphologies of the PBAT-Fe_3_O_4_@MWCNTs/Ni Microspheres and Composite Foams

**Fig. 2:**
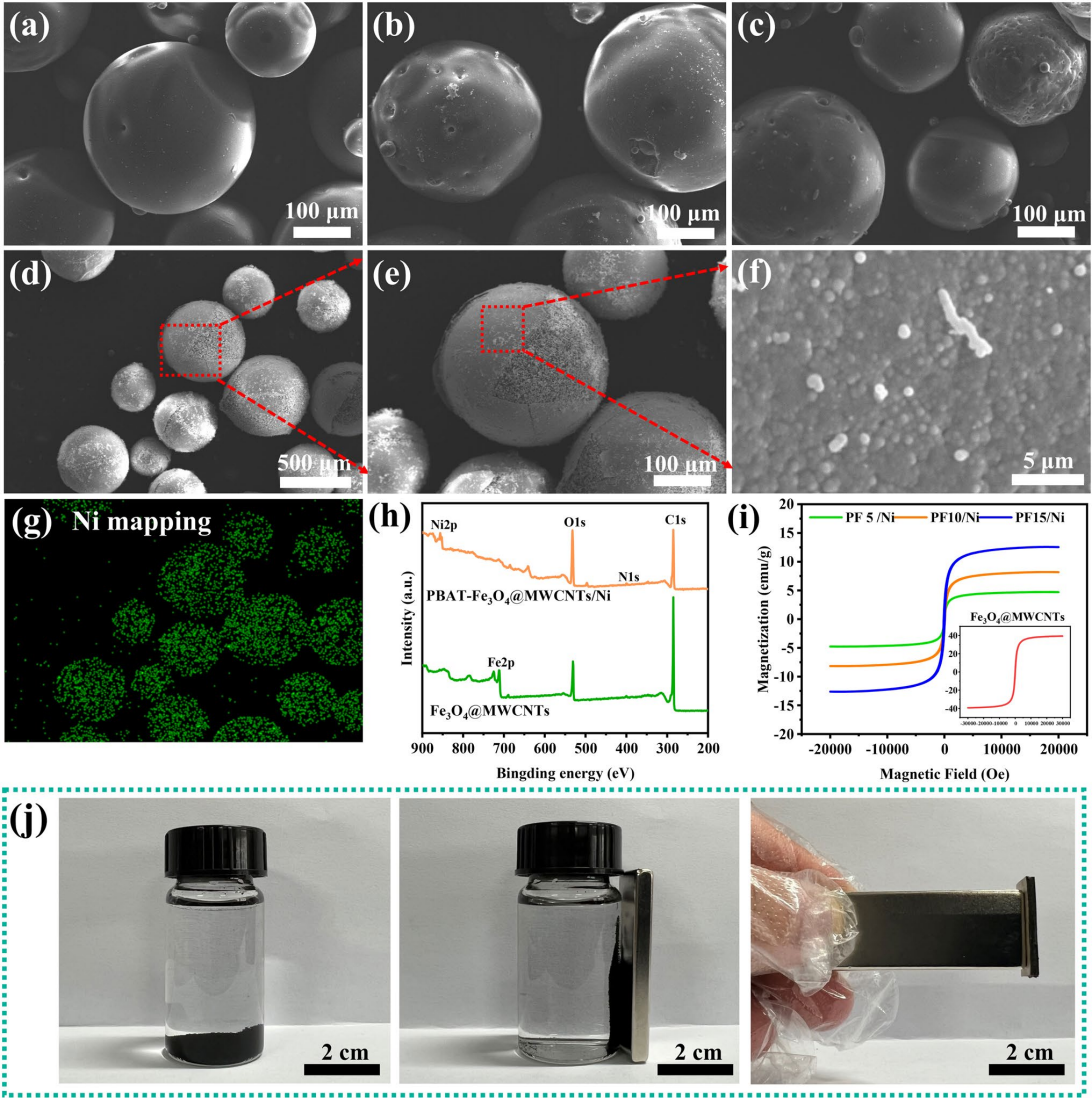
SEM images of PBAT-Fe_3_O_4_@MWCNTs microspheres containing **a** 5 wt%, **b** 10 wt% and **c** 15 wt% Fe_3_O_4_@MWCNTs; **d–f** Morphology of PBAT-Fe_3_O_4_@MWCNTs/Ni microspheres at different magnifications; **g** EDS mapping of the microspheres corresponding to **d**; **h** XPS spectra and **i** magnetization curves of Fe_3_O_4_@MWCNTs nanoparticles and PBAT-Fe_3_O_4_@MWCNTs/Ni microspheres; **j** Dispersion of microspheres in the absence and presence of magnetic field, response of sample under the action of magnet

Figure 2a–c display the morphology of PBAT-Fe_3_O_4_@MWCNTs microspheres with different filler contents. The prepared microspheres exhibit a regular spherical shape, which may contribute to the construction of isolated structural conducting network. Differing from the smooth surface of PBAT-Fe_3_O_4_@MWCNTs microspheres, a layer of micro-nanoparticles was attached to the surface of PBAT-Fe_3_O_4_@MWCNTs/Ni microspheres obtained after chemical deposition (Fig. 2d–e). The loaded particles were meticulously arranged in close proximity and uniformly dispersed, resulting in the formation of a compact, conductive layer that enhanced the convergence of conductive pathways (Fig. 2f). The EDS and XRD spectra of the microspheres reveal that the distribution of nickel elements is consistent with the microstructure of Fig. 2d, implying that the nickel nanoparticles are successfully encapsulated on the surface of PBAT-Fe_3_O_4_@MWCNTs microspheres (Figs. 2g and S1) [36, 37]. As a result, PBAT-Fe_3_O_4_@MWCNTs/Ni microspheres with core–shell structure were successfully obtained by solution blending and deposition. The uniform coating of nickel particles on the microsphere surface created a highly conductive layer, while Fe_3_O_4_@MWCNTs nanoparticles were dispersed in the interior of PBAT matrix, constituting a lossy core layer for EM waves [38, 39].

**Fig. 3:**
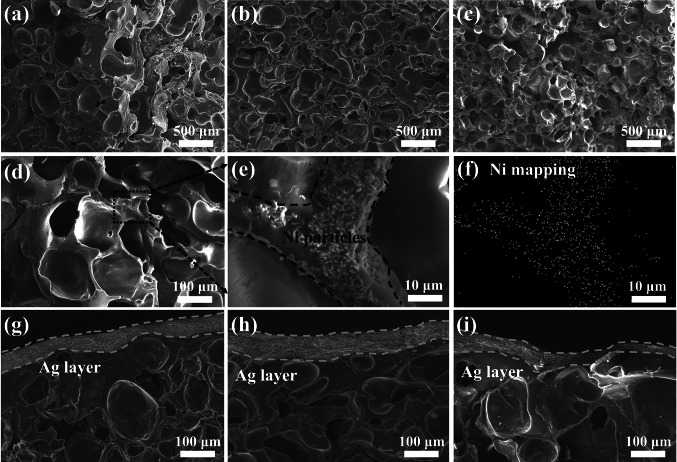
The SEM images of F-PF/Ni/Ag composite foams with **a** 5 wt%, **b** 10 wt% and **c** 15 wt% Fe_3_O_4_@MWCNTs; Dispersion of **d–f** Ni particles and **g–i** Ag layers in the composite foam

The surface chemical compositions of microspheres were further determined by XPS spectra. As shown in Fig. 2h, the peak of Fe 2*p* appearing in the XPS spectrum of PBAT-Fe_3_O_4_@MWCNTs/Ni microspheres coincided with the peak of Fe_3_O_4_@MWCNTs nanoparticles, and the position of the O 1*s* peak shifted from 532.08 to 531.08 eV, which was attributed to the formation of lattice oxygen (Fe–O) in Fe_3_O_4_ nanoparticles. The emergence of the Ni 2*p* peak unequivocally validated the successful encapsulation of nickel particles onto the surface of PBAT-Fe_3_O_4_@MWCNTs microspheres. The magnetization curves of the compound microspheres and magnetic particles are displayed in Fig. 2i. The saturation magnetization of pure Fe_3_O_4_@MWCNTs nanoparticles was 39.33 emu g^−1^, while the magnetic properties of the compound microspheres were improved with the addition of Fe_3_O_4_@MWCNTs. The saturation magnetization of the microspheres can reach about 12 emu g^−1^ at 15 wt% Fe_3_O_4_@MWCNTs. Moreover, the microspheres and composites could respond quickly under the application of magnets, and the favorable magnetic properties of the composites are beneficial for the enhancement of their EM wave absorption performance (Fig. 3j) [40, 41].

A porous structure was constructed to improve the absorption performance of the EMI shielding composites. The morphology of PBAT/Fe_3_O_4_@MWCNTs/Ni composite foam is presented in Fig. 3a–c, prepared by scCO_2_ foaming technique. Observable is the progressive reduction in cell size with the introduction of magnetic particles. This phenomenon is attributed to the pivotal role played by magnetic particles in nucleating cells as heterogeneous nucleation points during the foaming process. The heightened magnetic particle content contributes to an enhanced heterogeneous nucleation effect, consequently yielding a greater volume of pores in the final sample [25, 26, 42, 43]. The skeleton morphology of the composite foam is shown in Fig. 3d–f, where the metallic nickel particles can be detected. The elemental mapping analysis reveals the selective dispersion of nickel particles along the cell walls compared to the cross-sectional morphology of the solid sample (Fig. S2), promoting the establishment of interconnected pathways that effectively dissipate microwaves. Figure 3g–i reveal the SEM images of silver layer, which is attached to the top of the composite foam, and the adhesive resin in the silver gel guarantees a strong binding between the polymer substrate and silver particles. The foaming treatment reinforces the interfacial bonding between polymer particles through the secondary melting of microspheres under elevated temperature and pressure conditions.

### Electrical Conductivity and EMI Shielding Properties of the Solid and Foamed PBAT-Fe_3_O_4_@MWCNTs Composites

**Fig. 4:**
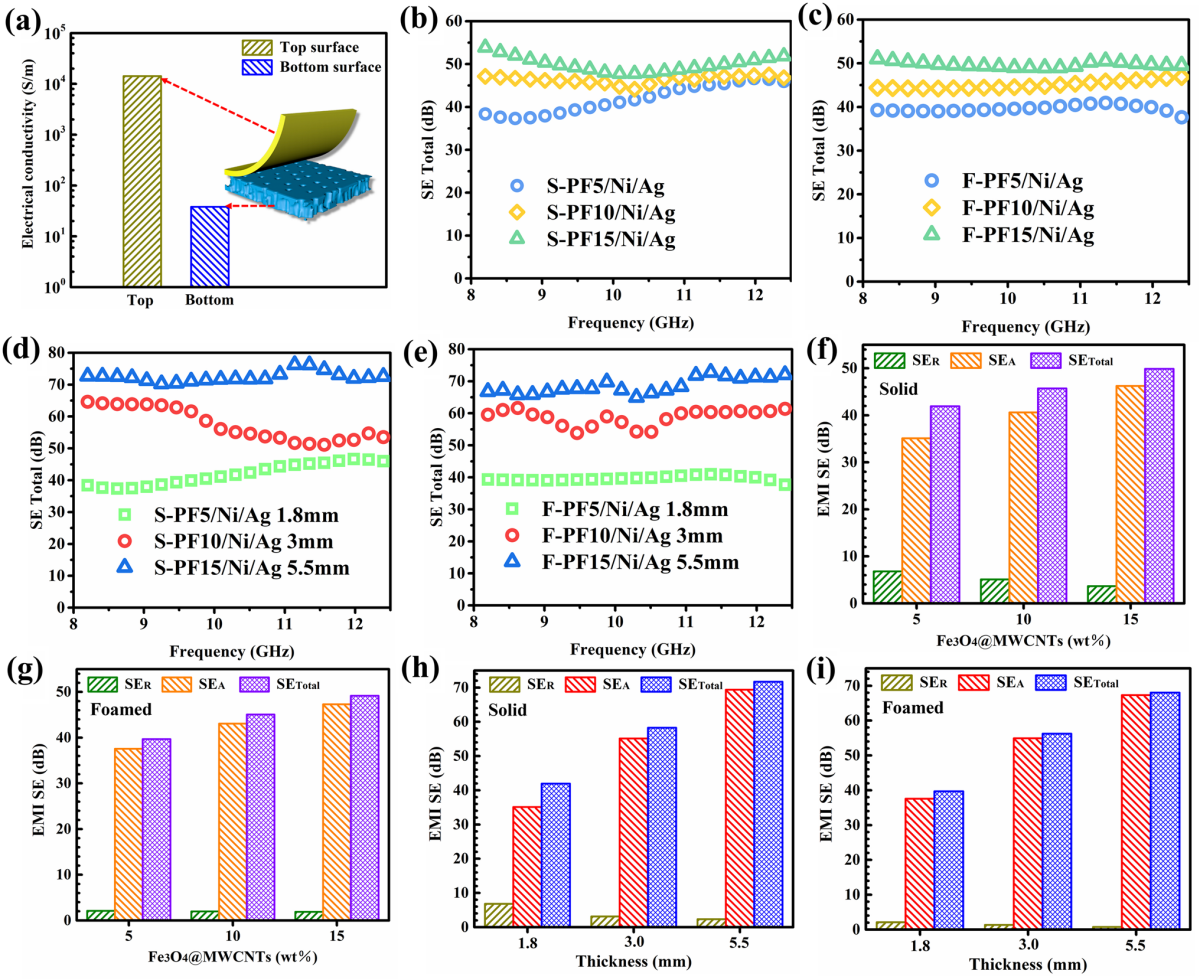
**a** Electrical conductivity of the top and bottom surfaces for F-PF15/Ni/Ag composite; EMI SE of S-PF/Ni/Ag and F-PF/Ni/Ag composites with different **b–c** Fe_3_O_4_@MWCNTs content and **d–e** thickness; Average SE values for **f** S-PF/Ni/Ag and **g** F-PF/Ni/Ag composites with different Fe_3_O_4_@MWCNTs content; Average SE values of **h** S-PF/Ni/Ag and **i** F-PF/Ni/Ag composites with different thickness

Electrical conductivity has been an essential characteristic for investigating the EMI shielding behavior of the composites [5, 10]. Figure 4a presents the comparative conductivity of the top and bottom surfaces of F-PF15/Ni/Ag composite. The prepared sample exhibits a layered structure, where the conductivity of the top surface can be up to 14,285 S m^−1^ thanks to the adhesion of highly conductive silver particles, while the bottom surface is composed of foamed microspheres with a conductivity of 38.1 S m^−1^. This special layered structure allows EM waves to penetrate inside the composite foam from the bottom surface with low conductivity, which is subsequently scattered and reflected by the highly conductive silver layer. Figure 4b–c performs the EMI SE of solid and foamed composites with a variety of Fe_3_O_4_@MWCNTs contents. In general, the EMI SE of shielding materials is required to be greater than 20 dB in commercial application environments [27, 44, 45]. The average EMI SE of the composites can reach 49.9 dB with a Fe_3_O_4_@MWCNTs content of 15 wt% due to the generation of a conducting network in the isolation structure. This is attributed to the high efficiency of connectivity network and the extensive presence of conductive interfaces for the honeycomb-shaped networks [46, 47]. Despite the disruption of the isolated structural network within the composites during the foaming process, the EMI SE value continues to surpass 45 dB in the X-band. This is chiefly ascribed to the exceptional conductivity of the surface silver particles within the layered structure, ensuring that the shielding efficiency does not experience a substantial decline.

The thickness of the sample exerts a discernible influence on the EMI shielding performance of the materials, typically exhibiting a positive correlation between the EMI SE of the composite and its thickness. The EMI SE of composites with different thicknesses is illustrated in Fig. 4d–e, and it can be seen that the EMI SE improves with the increase in thickness. When the sample thickness reaches 5.5 mm, the mean EMI SE of solid and foamed samples can reach 71.7 and 68.0 dB, respectively. This result is mainly attributed to the prolongation of the traveling path of incoming EM wave within the composite, which leads to enhanced interaction of the EM waves with the magnetic filler as well as the porous, thus improving the shielding properties. Moreover, the EMI SE of the foamed samples is marginally lower in comparison to that of the solid samples. This disparity can be attributed to the creation of a porous structure, which diminishes the concentration of magnetic particles and hampers the convergence of the conductive network (Fig. S3) [12, 25].

In order to better analyze the shielding mechanism of the composites, the reflected SE (SE_*R*_) and absorbed SE (SE_*A*_) were thus separated from the total SE (SE_Total_), and their variations with Fe_3_O_4_@MWCNTs content and sample thickness are displayed in Fig. 4f–g. The SE_*R*_ value of solid composite decreases with the addition of Fe_3_O_4_@MWCNTs due to the intense magnetic loss effect on the EM waves, which converts the microwaves into heat or other form of energy. The SE_*R*_ value could be obtained as low as 3.7 dB at 15 wt% Fe_3_O_4_@MWCNTs content. In addition, the foamed samples exhibit relatively lower SE_*R*_ values with regard to the solid samples, which diminishes the reflection loss. Figure 4h–i illustrates the influence of thickness on the SE_*R*_, SE_*A*_ and SE_*T*_ values, and it can be observed that the solid composite displayed a SE_*R*_ of 2.3 dB at 5.5 mm thickness. The SE_*R*_ of foamed sample decreases from 2.1 to 0.7 dB with an increasing thickness. The above results indicate that the increment of thickness can enhance the SE_*T*_ and SE_*A*_ values while simultaneously diminishing the SE_*R*_, and the layered networks constructed by highly conductive silver layers and magnetic Fe_3_O_4_@MWCNTs nanoparticles enable the dielectric and magnetic losses to be interacted with each other cooperatively, which combines the excellent EMI SE and low reflection [42, 48].

**Fig. 5:**
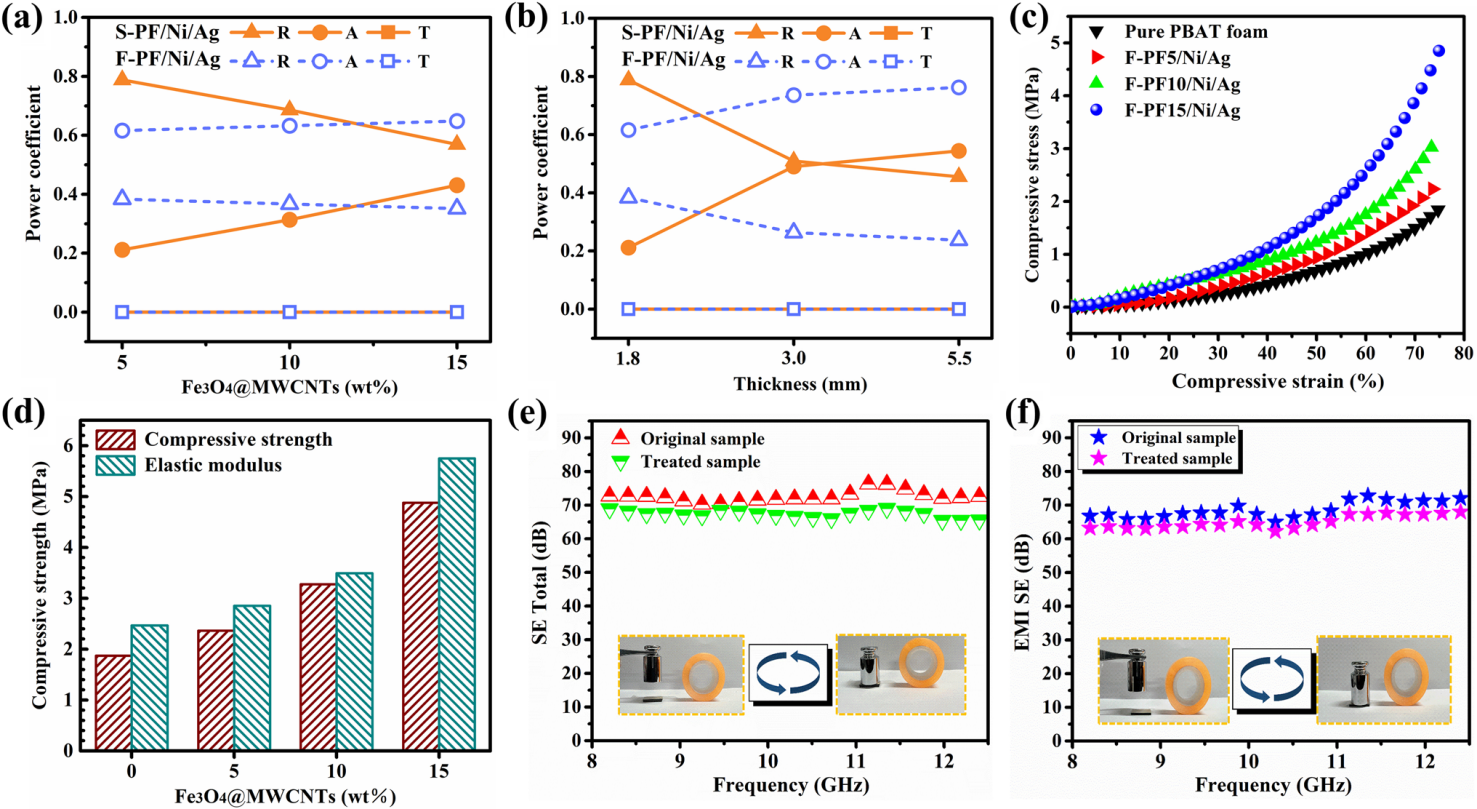
Power balance as function of **a** Fe_3_O_4_@MWCNTs content (1.8 mm) and **b** thickness (15 wt% Fe_3_O_4_@MWCNTs) for S-PF/Ni/Ag and P-PF/Ni/Ag composites; **c** Stress–strain curves, **d** compressive strength and modulus of F-PF/Ni/Ag composites in the compression test under a 75% strain; EMI SE of the **e** S-PF/Ni/Ag and **f** F-PF/Ni/Ag composites before and after a peeling experiment of 500 times under 100 g weight pressure

For the purpose of visually evaluating the dissipation ability of the layered structural composites, the *A*, *R* and *T* values of the solid and foamed composites were further investigated. As shown in Fig. 5a, the mean *R* value of solid sample (1.8 mm thickness) is 0.56 at 15 wt% Fe_3_O_4_@MWCNTs content, while the *R* value of the foamed group decreases with the addition of magnetic particles, and the *R* value is only about 0.35 with 15 wt% Fe_3_O_4_@MWCNTs. On the one hand, Fe_3_O_4_@MWCNTs can improve the magnetic loss of the samples by increasing their residual loss and hysteresis loss thus augmenting the dissipation of the incident EM waves [9, 11, 49]. On the other hand, EM waves will be attenuated and absorbed significantly as they travel through the micro-porous structure composite. Upon reaching the opposite side, the microwave undergoes another round of reflection from the exceptionally conductive silver layer, subsequently being re-absorbed. The deliberate engineering of the impedance matching layer and the highly conductive layer engenders a distinctive “absorption-reflection-re-absorption” sequence, ultimately dispersing the microwave energy as heat or otherwise. This process offers an efficient means to mitigate secondary pollution. The effect of thickness on the power coefficient is given in Fig. 5b, the solid composite presents an average *R* value of 0.45 with 5.5 mm thickness. However, the R value can be reduced to 0.23 by scCO_2_ foaming, which implies an absorptivity as high as 77% for the foamed composite. This is because the increased thickness enables the EM waves to propagate in an extended path within the composite and be progressively attenuated as much as possible. The fabricated composites exhibit an absorption-dominated shielding mechanism.

### Compression Properties and EMI Shielding Stability of the PBAT-Fe_3_O_4_@MWCNTs/Ni/Ag Composite Foams

The stress–strain curves and compressive strength of the composite foams are presented in Fig. 5c–d at different Fe_3_O_4_@MWCNTs contents. The introduction of nanoparticles led to a remarkable enhancement in the compressive properties of the samples. The compressive strength and compressive modulus of the composite foams rises from 1.87 and 2.46 MPa to 4.88 and 5.75 MPa, respectively. This outcome can primarily be ascribed to the augmented cell density and reduced cell diameter resulting from the higher concentration of Fe_3_O_4_@MWCNTs particles. In general terms, an increased quantity of cell prisms and thicker cell walls can improve the compressive resistance of the composite foam within the same volume [50]. Furthermore, the addition of filler particles also plays a role in reinforcing the matrix to bear the external pressure. Theoretically, the platform region is caused by the collapsing of the bubbles. The platform area on the stress–strain curve for the prepared composite foam is relatively limited, indicative of a well-structured network formation within the foam that imparts superior compressive capacity.

Beyond that, both the solid and foamed samples retained their commendable shielding properties even after undergoing tape peeling experiments for 500 times under 100 g weight pressure (Fig. 5e–f). The retention of the EMI SE for solid composite is as high as 94.6% in the X-band, which suggests an excellent adhesion between the silver layer and the substrate. This can be primarily attributed to the curing of the adhesives, including 2-Propenoic acid and certain homopolymers, present in the silver paste, which ultimately establishes a robust connection between the silver particles and PBAT substrate.

### EMI Shielding Mechanism of the PBAT-Fe_3_O_4_@MWCNTs/Ni/Ag Composite Foams

**Fig. 6:**
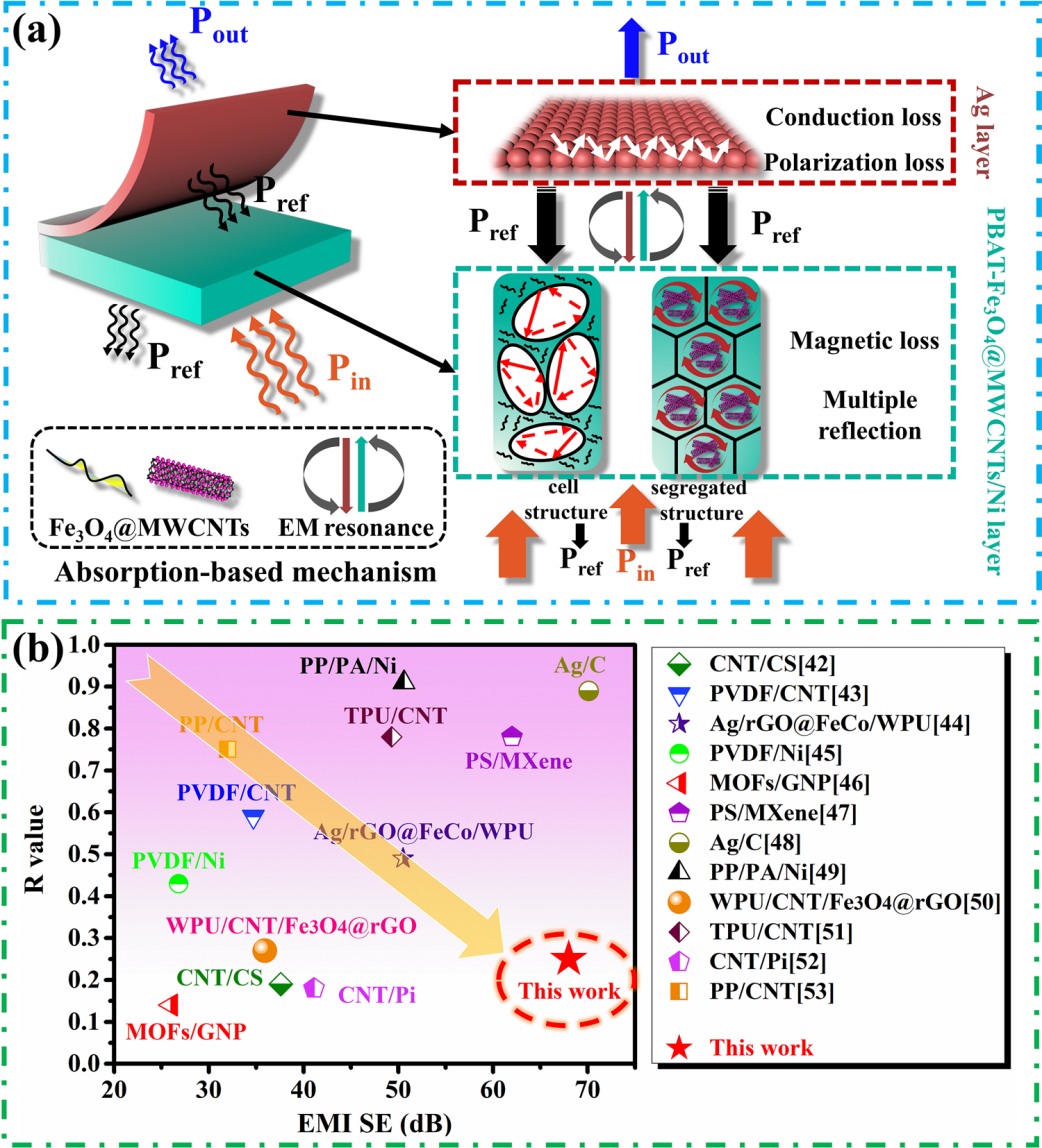
**a** Scheme of EM wave dissipation mechanism within the composite foams; **b** Comparison of EMI SE and R value of this work and previously reported EMI shielding materials

Figure 6a demonstrates the shielding mechanism of the layered structural composite foam constructed by the isolated network, microcellular and silver layer. While EM waves enter into the interior of the composite, they were absorbed through conductive loss, dielectric loss and hysteresis loss. Additionally, the interfaces available between the conductive networks contribute to the multiple reflection and attenuation of the microwaves, which is ascribed to the synergistic effect of the magnetic particles and nickel layer. The isolated networks serve to introduce an extensive array of conducting surfaces via the honeycomb architecture, thereby elongating the transmission path of EM waves and intensifying their scattering through repetitive internal reflection. Simultaneously, the porous structure diminishes impedance mismatch and engenders numerous air-matrix boundaries, effectively entrapping microwaves and causing them to undergo multiple rounds of reflection and absorption within the composite. The majority of the remaining microwaves are reflected when the EM wave reaches the highly conductive silver layer and re-absorbed again by traveling via the isolated and porous PBAT-Fe_3_O_4_@MWCNTs/Ni composite foam. The reasonable integration of the above impedance matching layer and highly conductive layer constitutes such a special “absorption-reflection-re-absorption” procedure. Nearly, 99.99999% of the incident EM waves were blocked, and most of them were absorbed instead of being reflected to the external space, revealing a highly efficient and low-reflection EMI shielding properties. Accordingly, the EMI shielding performance of PBAT-Fe_3_O_4_@MWCNTs/Ni/Ag composite foam is superior to the recently reported EMI shielding materials (Fig. 6b) [51–62], attributed to this particular structural design and shielding mechanism.

## Conclusion

In summary, the layered structural PBAT-Fe_3_O_4_@MWCNTs/Ni/Ag composite foams with high shielding efficiency and low reflection were developed by the integrated design of isolated network and scCO_2_ foaming. The isolated conductive network is composed of the overlapping nickel particles on the PBAT-Fe_3_O_4_@MWCNTs microspheres synthesized by phase separation, while the silver layer attached on the top surface of composite contributes to the uniquely layered structure. The Fe_3_O_4_@MWCNTs nanoparticles and microcellular structure facilitate magnetic loss and multiple reflective absorption of EM waves, thereby augmenting microwave dissipation. Simultaneously, the silver particle layer predominantly governs dielectric loss. The above-mentioned effects integrate multiple loss modes organically and result in an “absorption-reflection-re-absorption” EMI shielding mechanism. The findings indicate that the proposed composite foam can attain an average EMI SE of 68.0 dB with an impressively low reflectivity of just 23%. Moreover, these composite foams exhibit robust EMI shielding performance even after undergoing 500 cycles of adhesion experiments. This work offers an ingenious strategy to the development of EMI shielding materials with reliable high absorptivity and efficient EMI SE properties.

## Supplementary Information

Below is the link to the electronic supplementary material.

Supplementary file1 (PDF 621 KB)
