# Supplementary material for: Layered Structural PBAT Composite Foams for Efficient Electromagnetic Interference Shielding
Source: Nanomicro Lett. 2023 Nov 23;16:31. doi: 10.1007/s40820-023-01246-8 (PMC10667195; doi:10.1007/s40820-023-01246-8)
Supplement: Supplementary file 1 — Supplementary file1 (PDF 621 KB) [file 40820_2023_1246_MOESM1_ESM.pdf]

Supporting Information for

Layered Structural PBAT Composite Foams for Efficient Electromagnetic Interference Shielding

Jianming Yang[1, 3], Hu Wang[1], Yali Zhang[2], Hexin Zhang[1, *], Junwei Gu[2, *]

[1]School of Chemistry and Chemical Engineering, Anhui University of Technology, Ma'anshan, Anhui 243032, P. R. China

[2]Shaanxi Key Laboratory of Macromolecular Science and Technology, School of Chemistry and Chemical Engineering, Northwestern Polytechnical University, Xi'an, Shaanxi 710072, P. R. China

[3]School of Materials Science and Engineering, Jiangsu University of Science and Technology, Zhenjiang, Jiangsu 212003, P. R. China

*Corresponding authors. E-mail: hxzhang@ahut.edu.cn (H. Zhang); gjw@nwpu.edu.cn or nwpugjw@163.com (J. Gu)

S1 Characterization Section

The X-ray diffraction was performed by an X-ray diffractometer (XRD, Bruker D8 Advance, Germany) with a Cu·Kα radiation source (40 kV and 30 mA). Chemical composition of microspheres was analyzed by an X-ray photoelectron spectroscopy (XPS, Dmax2500, Rigaku, Japan). Magnetic properties of Fe_3O_4@MWCNTs were characterized by a vibrating sample magnetometer (VSM, Lakeshore7404, USA) at 300 K. Scanning electron microscope (SEM, Nano SEM 430, FEI, USA) was employed to observe the microstructure of the microspheres and composites at an accelerating voltage of 5 kV. Energy dispersive X-ray spectrometry (EDS, X-Max Extreme, Oxford, UK) mapping was carried out to analyze the dispersion of Ni. The electrical conductivity of the composites was determined using a four-point probe apparatus (RTS-9). EMI shielding performance was assessed with an Agilent N5230 vector network analyzer (USA) according to ASTM ES7-83 standards. The shape of the sample is a disk with a diameter of 13 mm. The total SE (SE_T), reflected SE (SE_R), absorbed SE (SE_A), reflection coefficient (R), transmission coefficient (T) and absorption coefficient (A) can be calculated as follows:

$$R = |S_{11}|^2 \tag{S1}$$

$$T = |S_{21}|^2 \tag{S2}$$

$$A = 1 - R - T \tag{S3}$$

$$SE_T = -10\log T \tag{S4}$$

$$SE_R = -10\log(1 - R) \tag{S5}$$

$$SE_A = -10\log\left(\frac{T}{1-R}\right) = SE_T - SE_R - SE_M \tag{S6}$$

Where the multiple reflection (SE_M) could be ignored when SE_T >15 dB [S1-S3].

S2 XRD Patterns of Fe_3O_4@MWCNTs Nanoparticles and PBAT-Fe_3O_4@MWCNTs/Ni Microspheres

Figure S1 exhibits the XRD spectra of Fe_3O_4@MWCNTs and PBAT-Fe_3O_4@MWCNTs/Ni microspheres, and it can be seen that the Fe_3O_4@MWCNTs nanoparticles display five distinctive characteristic peaks corresponding to (220), (311), (400), (511) and (440) crystal planes, which align with the cubic anti spinel structure of magnetite (JCPDS CARD NO. 19-0629). Meanwhile, the prepared PBAT-Fe_3O_4@MWCNTs/Ni microspheres also exhibit similar XRD curve characteristics. Moreover, the three typical characteristic peaks at 2θ = 44.92°, 51.06° and 76.48° are in conformity with the (111), (200) and (220) crystal plane diffractions of Ni, which are consistent with the standard face-centered cubic structure of Ni metal (JCPDS no. 04-08507) [S4-S5], indicating that the Ni particle layer has been deposited on the surface of PBAT-Fe_3O_4@MWCNTs microspheres.

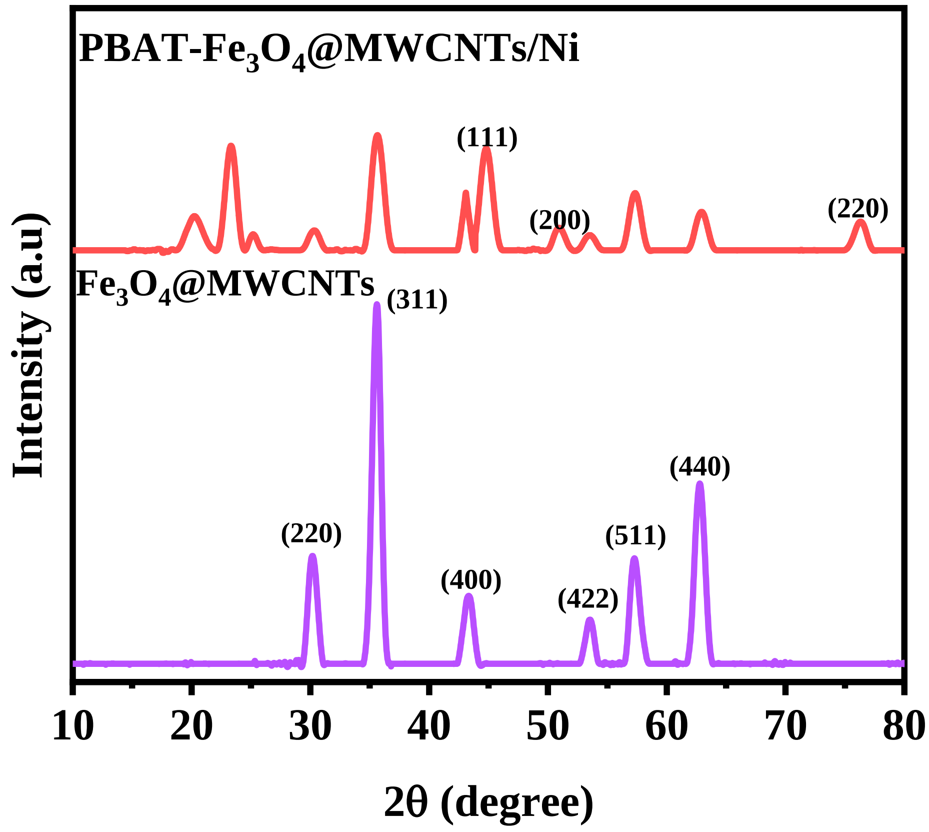

Fig. S1 XRD patterns of Fe_3O_4@MWCNTs nanoparticles and PBAT-Fe_3O_4@MWCNTs/Ni microspheres

S3 SEM Images of Isolated PBAT-Fe_3O_4@MWCNTs/Ni Composites

The cross-sectional micromorphology of PBAT-Fe_3O_4@MWCNTs/Ni composites is presented in **Fig. S2**, where the microspheres were deformed into polygonal shapes because of the high-intensity extrusion during the molding process (**Fig. S2a-b**). Ni particles are concentrated at the interface of microspheres, establishing interconnections that give rise to a complex three-dimensional isolated network structure (**Fig. S2c-d**). High pressure and low temperature molding conditions were adopted in the fabrication of isolated structural composites to prevent the filler from diffusing into the interior of polymer matrix [S6-S7]. The presence of defects was not observed by local magnification of SEM images, ascertaining the strong bonding between the Ni particles and PBAT-Fe_3O_4@MWCNTs microspheres. This configuration proves highly advantageous for the construction of an interfacial skeleton that facilitates multiple reflections and absorptions of EM waves. This results primarily from the deliberate filler distribution at the micro-phase interface [S8-S10].

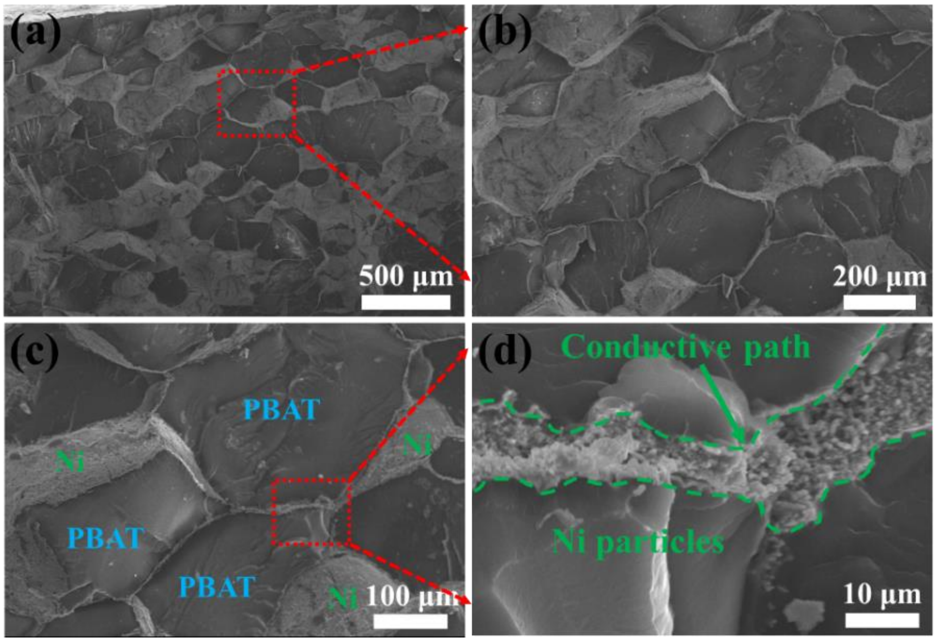

Fig. S2 a-d SEM images of the cross-section for isolated PBAT-Fe_3O_4@MWCNTs/Ni composites at different magnification

S4 Conductivity and Density of the Solid and Foamed PBAT-Fe_3O_4@MWCNTs/Ni/Ag Composites

As illustrated in **Fig. S3**, the conductivity of both solid and foamed PBAT-Fe_3O_4@MWCNTs/Ni/Ag composites was significantly enhanced with the addition of Fe_3O_4@MWCNTs nanoparticles. This is due to the synergistic overlap of the nanoparticles inside the PBAT microspheres with the Ni particles at interface, which strengthens the three-dimensional conductive network structure. In addition, the

introduction of porous structure reduces the electrical conductivity of the composites resulting from the disruption of original isolated network consisting of Ni particles, which in turn alleviates the impedance mismatch between the composites and air, facilitating the multiple reflections of EM waves incident inside the composites through the microporous (**Figs. 4g** and **5a**).

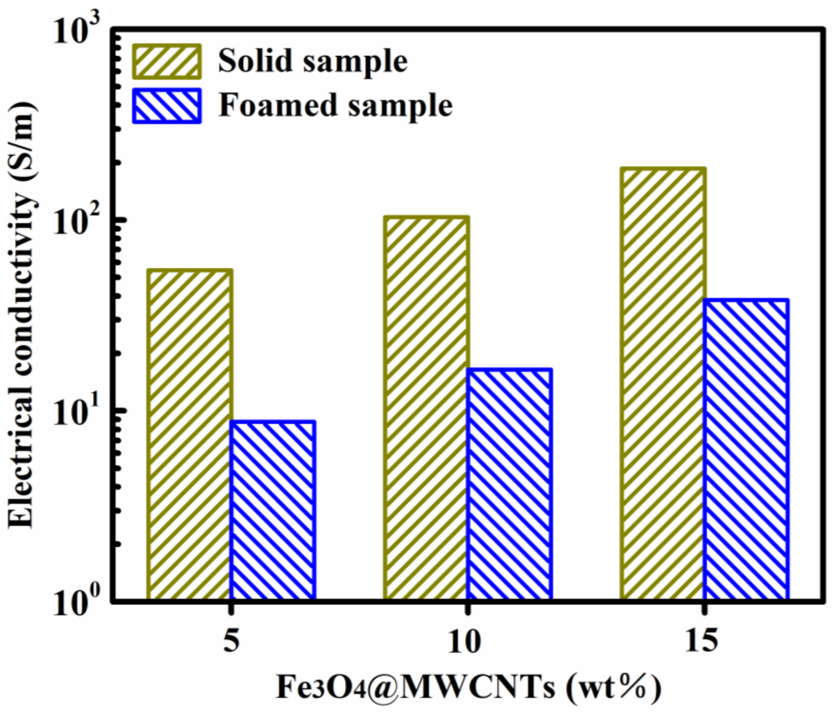

Fig. S3 The conductivity of the bottom surfaces of solid and foamed PBAT-Fe_3O_4@MWCNTs/Ni/Ag composite with various filler contents

The EMI shielding behavior of the composite foams were also affected by their density. As the content of Fe_3O_4@MWCNTs was raised from 5 to 15 wt%, the density of the solid composite increased from 1.319 to 1.722 g/cm^3. The density of the samples can be reduced by approximately 50% after scCO_2 foaming (**Fig. S4**). Such a significant weight reduction not only helps to broaden the application area for EM shielding materials, but also offers efficient absorption and dissipation of EM waves through the air-substrate interface in a large amount of porosity.

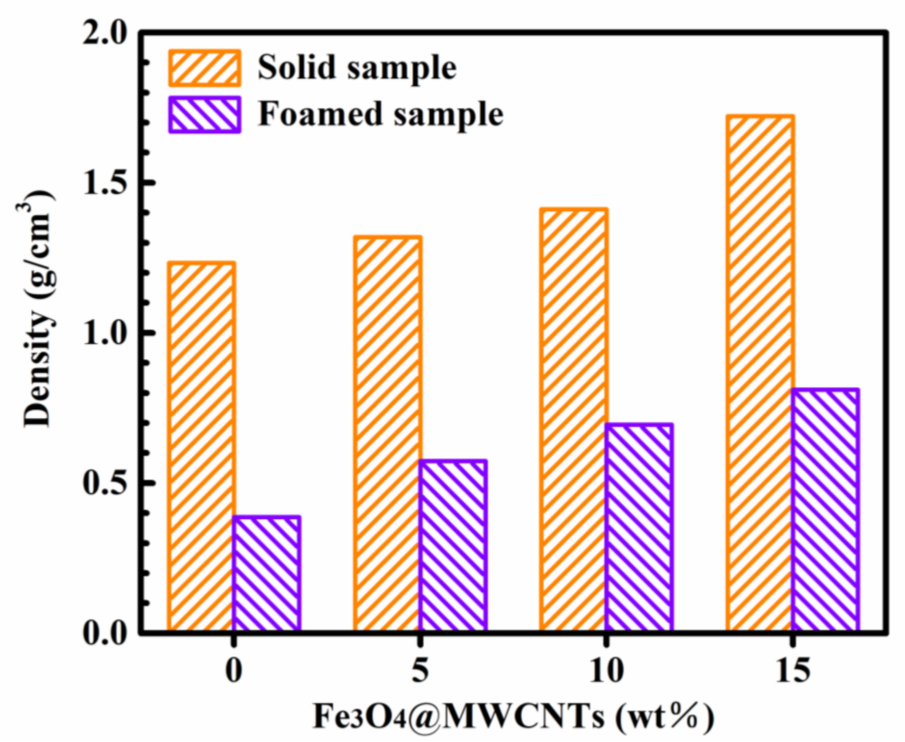

Fig. S4 Density of solid and foamed composites with different Fe_3O_4@MWCNTs content

Supplementary References

[S1] Z. Lei, D. Tian, X. Liu, J. Wei, K. Rajavel et al., Electrically conductive gradient structure design of thermoplastic polyurethane composite foams for efficient electromagnetic interference shielding and ultra-low microwave reflectivity. Chem. Eng. J. **424**, 130365 (2021). https://doi.org/10.1016/j.cej.2021.130365

[S2] J. Chen, X. Liao, W. Xiao, J.M. Yang, Q.Y. Jiang et al., Facile and green method to structure ultralow-threshold and lightweight polystyrene/MWCNT composites with segregated conductive networks for efficient electromagnetic interference shielding. ACS Sustain. Chem. Eng. **7**, 9904–9915 (2019). https://doi.org/10.1021/acssuschemeng.9b00678

[S3] Z.Y. Jiang, Y.J. Gao, Z.H. Pan, M.M. Zhang, J.H. Guo et al., Pomegranate-like ATO/SiO_2 microspheres for efficient microwave absorption in wide temperature spectrum. J. Mater. Sci. Technol. **174**, 195–203 (2024) https://doi.org/10.1016/j.jmst.2023.08.013

[S4] Q. Qi, L. Ma, B. Zhao, S. Wang, X. Liu et al., An effective design strategy for the sandwich structure of PVDF/GNP-Ni-CNT composites with remarkable electromagnetic interference shielding effectiveness. ACS Appl. Mater. Inter. **12**, 36568–36577 (2020). https://doi.org/10.1021/acsami.0c10600

[S5] J.M. Yang, Y.J. Chen, X. Yan, X. Liao, H.X. Zhang et al., Construction of in-

situ grid conductor skeleton and magnet core in biodegradable poly (butyleneadipate-co-terephthalate) for efficient electromagnetic interference shielding and low reflection. Compos. Sci. Technol. **240**, 110093 (2023). https://doi.org/10.1016/j.compscitech.2023.110093

[S6] D.X. Yan, H. Pang, B. Li, R. Vajtai, L. Xu, P.G. Ren, Z.M. Li et al., Structured reduced graphene oxide/polymer composites for ultra-efficient electromagnetic interference shielding. Adv. Funct. Mater. **25**, 559–566 (2015). https://doi.org/10.1002/adfm.201403809

[S7] L.C. Jia, D.X. Yan, C.H. Cui, X. Jiang, Z.M. Li et al., Electrically conductive and electromagnetic interference shielding of polyethylene composites with devisable carbon nanotube networks. J. Mater. Chem. C **3**, 9369–9378 (2015). https://doi.org/10.1039/C5TC01822F

[S8] J.M. Yang, Y.J. Chen, C. Liu, X.Z. Chai, Z.P. Chen et al., Constructing 3D expanded graphite-silver segregated network structure for ultra-efficient EMI shielding and low reflection. J. Mater. Res. Technol. **23**, 5115–5126 (2023). https://doi.org/10.1016/j.jmrt.2023.02.105

[S9] Y. Zhan, J. Wang, K. Zhang, Y. Lia, Y. Meng et al., Fabrication of a flexible electromagnetic interference shielding Fe_3O_4@reduced graphene oxide/natural rubber composite with segregated network. Chem. Eng. J. **344**, 184–193 (2018). https://doi.org/10.1016/j.cej.2018.03.085

[S10] R. Sun, H. Zhang, J. Liu, X. Xie, R. Yang et al., Highly conductive transition metal carbide/carbonitride (MXene)@polystyrene nanocomposites fabricated by electrostatic assembly for highly efficient electromagnetic interference shielding. Adv. Funct. Mater. **27**, 1702807 (2017). https://doi.org/10.1002/adfm.201702807
